# The expansion of vanity of Simon in A Portrait of the Artist as a Young Man

**DOI:** 10.3389/fpsyt.2023.1187171

**Published:** 2023-05-22

**Authors:** Hamid Farahmandian, Chaojian Liu

**Affiliations:** ^1^School of Foreign Languages, Chengdu University, Chengdu, China; ^2^School of International Communications & Education, Communication University of Zhejiang, Hangzhou, China

**Keywords:** James Joyce, Karen Horney, neurosis 4, *A Portrait of the Artist as a Young Man*, vanity

## Abstract

An aggressive personality is a distorted personality with dark traits such as arrogance, a sense of power over others, and exploitation. As per Karen Horney's theory of neurosis, all these traits make an individual, psychologically, neurotic, who is willing to go against other people in society. In this paper, drawing upon Horney's theory, I study the aggressive personality of Simon in James Joyce's *A Portrait of the Artist as a Young Man* from three aspects of frustrated selfishness, sense of dominance, and search for respect to make known his neurotic needs for power, admiration, prestige, exploitation and achievement and demonstrate that offensive conducts of Simon make him more insecure and leave him more aggressive toward everybody at home and society.

## Introduction

Arrogance is the byproduct of vanity and deep-seated insecurities. Vanity, in conventional parlance, is the excessive belief in one's own attractiveness or abilities to others. Furthermore, in philosophy, vanity refers to a broader sense of pride and egoism as Friedrich Nietzsche believes that “vanity is the fear of appearing original: it is thus a lack of pride, but not necessarily a lack of originality” [(1), p. 168]. The definition of arrogance is in parallel with Karen Horney's (1885–1952) definition of aggressive personality as one of the three types of neurotics in which, a neurotic person “moves against people.” In this theory, the need for power, exploitation, Prestige, admiration, and achievement shape an aggressive personality. The individuals who move against other people need to survive in a competitive society: “The aggressive type takes it for granted that everyone is hostile, and refuses to admit that they are not; to him life is a struggle of all against all, and the devil take the hindmost [(2), p. 65]. This sort of neurotic people's needs originate from their feeling that the world is a place where, in the Darwinian sense, only the fittest survives and the strong obliterates the weak. While looking for help and approval of others, an aggressive person might also look for controlling them and decrease the threat and model of normality that others represent. Horney emphasizes that individuals with aggressive personalities enjoy their vanity and narcissism because they cannot tolerate any reaction from others to their behaviors and failures. Moreover, they are interested in attracting others' attention by means of power and vanity: “they feel the keeping of appointments as intolerable coercion, they secretly enjoy making people wait; in addition, seeing no flaw in themselves, they put the blame on others or on untoward circumstances” [(2), p. 175]. Therefore, aggressive people are neurotic as they need to be flattered and in power.

In this paper, I aim to question James Joyce's (1882–1941) characterization of Simon which I believe to be more neurotic. While several researches [e.g., (3–7)] have been done to show the state of the perversion in Joyce's works, the idea of neurosis, which I aim to study, has been quite overlooked despite its significance in Joyce's characterization. Moreover, while most of the researches are drawn upon Jacques Lacan and Sigmund Freud's theories, I base my study on Horney's theory of neurosis, in this paper. To put forward my idea, I select Simon Dedalus, the father of Stephen Dedalus, from (8) as a case study. Simon has an aggressive personality due to his vanity and narcissism, which makes him move against other people and have an aggressive personality. Joyce illustrates that Simon's aggressiveness results from his failures in both society and private life. He desires to be at the center of public attention; however, he fails to satisfy his vanity due to the loss of others' admiration. Therefore, I will study the aggressive vanity of Simon from three aspects of frustrated selfishness, sense of dominance, and his search for respect.

## Simon's frustrated selfishness

Selfishness is concerned exclusively or excessively with oneself who seeks or concentrates on one's own wellbeing, pleasure or advantage without regard for others. An individual with an aggressive personality finds that being selfish, because of aggressiveness, makes him/her more attractive to other people. “The healthy individual will strive to overcome his or her inferiority through involvement with society. One is concerned about the welfare of others as well as oneself and develops good feelings of self-worth and self-assurance” [(9), p. 17]. Moreover, some people are more involved in selfishness rather than social interests. They might utter selfishness through a desire to dominate, to take and not to give, and to decline to collaborate: “From these unhealthy responses, the person develops an inferiority complex or a superiority complex” [(9), p. 18]. The people, like Simon in *A Portrait of the Artist as a Young Man*, with the superiority complex have hidden doubts about their abilities and skills and express the sense of selfishness obviously. Simon's selfishness makes him move against other people in order to achieve his own goals. He struggles to attract others' attention since he is anxious about his loneliness and helplessness. However, he is not successful in this due to his sense of selfishness. Therefore, being frustrated, Simon remains aggressive and goes neurotic in behavior. Joyce makes Simon's selfishness evident by his job preferences, his need for public privileges, and his self-interest at home.

At first, the selfishness of an individual can influence his tendency to accept a job position, which is below his current social level. In addition, as mentioned before, aggressiveness may impose neurotic individuals to see themselves as the winners of any competition in society. Therefore, it is impossible for them to accept any social rank lower than their current one. In *A Portrait of the Artist as a Young Man*, Simon used to live a wealthy life with a prosperous job; however, he has an ongoing worse financial situation currently. He needs a job to afford his family's expenses; however, he rejects to accept any job offers, which are lower than his previous positions. He has experienced numerous temporary jobs but unable to be fixed in any of them: “medical student, an oarsman, a tenor, an amateur actor, a shouting politician, a small landlord, a small investor, a drinker, a good fellow, a story-teller, somebody's secretary, something in a distillery, a tax-gatherer, a bankrupt and at present a praiser of his own past” [(8), p. 301]. One of the unfortunate consequences of Simon's irresponsible nature is that the family is often in financial difficulty. Moreover, as the publican progresses further downhill after Parnell's demise, these difficulties become more frequent. Although the loss of his previous job is not directly related to Parnell's fall, Simon worships “the uncrowned king of Ireland” [(10), p. 382] and blames his loss on anti-Parnell forces like the Roman Catholic Church. In addition, “Mr. Dedalus seems to know all the ‘right people' in Dublin, but it appears that they know him too well to trust him with any major responsibilities” [(11), p. 20]. As a selfish man, he will not settle for any job beneath his situation and fabricate a vision of himself as persecuted by those in power. Simon believes that he deserves the best of the bests; however, he goes blind to understand the current situation of his family, which has changed dramatically compared to the past. Besides, Simon seems improbable to perform or accept any job properly if he were offered one because of his vanity on one hand and his laziness on the other hand. It is obvious that Simon had better off days in the past, so he is used to an easy life. He prefers enjoying his own life to bearing his responsibilities toward his family. Simon does not intend to find a job; while he blames society, the government, and his own family for his unemployment and worse financial situation. Simon would be happy if his son, Stephen, could grow up properly to find a suitable job to help his family financially. Hence, selfish people cannot accept seeing other peers and people be better than them. They are in the fight with others to prove their vanity; while they end up in helplessness. In *A Portrait of the artist as a young Man*, selfishness and laziness have major influences on the characters' inability to face their real self. He is now selfish enough to think about himself rather than his family. Simon's selfishness makes him become arrogant and move against others.

Secondly, the need for more public privileges can be a major reason for selfishness. Aggressive people typically desire much more public privileges than other people. Privileges operate on institutional, cultural, interpersonal, and personal levels and give benefits, favors, and advantages to members of dominant groups. “Privileges are normally invisible to individuals who possess them. People in dominant groups often believe that they have earned the privileges that they enjoy or that everyone can obtain those privileges if only they work hard to earn them” [(12), p. 2]. In fact, privileges are unearned, and they are usually granted to people in the dominant groups whether they want those privileges or not regardless of their affirmed intents. However, individuals with aggressive personalities neurotically believe that they deserve more privileges than others, even if they do not work hard for them. Simon used to have access to numerous public privileges due to his social position. Nevertheless, he has been unsuccessful and irresponsible to keep them forever. Joyce discloses that Simon's aggressive personality in his struggle with society facilitates him to earn more benefits than he should have got. Simon believes that his current financial and social situation does not match his past successes and achievements. It is apparent that Simon does not intend to quit his past happy days full of fame, which have drowned him into a personality of extreme selfishness.

The great disappointment of his life was accentuated by a lesser and keener loss—the loss of a coveted fame. On account of a certain income and of certain sociable gifts Mr. Dedalus had seemed accustomed to regard himself as the center of a little world, the darling of a little society. This position he still strove to maintain but at the cost of a reckless liberality from which his household had to suffer, both in deed and in spirit [(8), p. 110].

It is obvious that Simon moves against people as he wishes to reclaim his lost privileges in society. We know that he had better days, and clung to the past, Simon was a successful and popular person. Instead of cleaning up his act, he sinks lower and lower into poverty and despondency, taking his family with him. He is particularly bogged down by his bitter life in the political state of Ireland. Simon is a believer in Parnell and in Irish Home Rule, but he has become disenchanted by the current situation. Under the past political situation, Simon used to be famed and of high social position; though, he loses all his privileges after the political changes in the country. In fact, Simon aggressively needs his past days to be proud of; however, he is unable to retain those advantages. He cannot recover himself from the damages of political alterations. The loss of social privileges and fame removes Simon's vanity and selfishness. Nevertheless, He still looks for opportunities to get back those missing privileges. The loss of privileges turns Simon into a radical selfish person who moves against other people in the struggle for personal benefits.

Thirdly, addicting to alcohol while a family is suffering from financial troubles is a high demonstration of a person's selfishness. Self-interest refers to the fact of somebody considering only their own interests and not caring about things that would help other people. A number of psychological, sociological, and philosophical theories examine the role of self-interest in relation to human behaviors. In other words, selfish people mostly look for their own interests and are indifferent to others'. They can easily fight to secure their interests at any cost. One of the reasons for Simon's self-interest in *A Portrait of the Artist as a Young Man* is alcoholism. Alcoholism is a “multifaceted physiological illness, with a probable genetic component, inflected by gender, social class, and milieu, and expressed both within and outside of cultural norms” [(13), p. 233]. Indifference to his family's financial problems, Simon enjoys his time with alcoholism. It is evident that Simon's personal and social failures are inextricable from alcoholism done within the norms of a particular patriarchal society. Joyce represents the portrait of Simon as a person who has ruined himself and, instead of facing his problems, drowns them in alcohol and prejudice. Simon's denial that his life has been ruined by alcoholism results in his hallucinated selfishness. While Naremore sees finances as Simon's aggressiveness and problems, Stephanie Brown adds alcoholism as a significant reason for Simon's selfishness. Brown (14) believes “that alcohol is a means to cope with something else that is identified as the major problem is often one proof of denial in an alcoholic family” (p. 35). Simon has begun drinking even more heavily than he used to and his family life is in utter disarray. He spends the little money he has on drinking instead of feeding his children. Simon's only interest is using all the money he can to stand himself and others round of drinks at the bar. He is desperate not only to defend his right to drink but to hide that drinking is his intention. Simon's arrogance and subterfuge in protecting his right to drink are evident. “To those familiar with the effect of alcoholism on families, it is not ahistorical after studying this scene to conclude that Simon Dedalus is an alcoholic whose drinking has degraded his promising life and has forced his family into extreme poverty” [(13), p. 157].

Furthermore, one of the clear results of alcoholism is depression. Consideration of alcoholism as a trauma signifies depression because of loss of control and consequent loss of self-esteem and seriously disturbs interpersonal relationships. Simon's loss of control after each time being drunk at home or pub makes him overlook other people around in a way that he even becomes disrespectful to his own son in front of other strange people. His son, Stephen is ashamed of his father's drinking and flirtation with the barmaids. When Simon is sober, he emotionally agonizes about his nostalgic past; however, when he is drunk, he loses his own consciousness and enjoys his time in an embarrassing way. Thus, alcoholism and addiction to alcoholism can make an individual selfish enough to think about his own situation. Joyce represents Simon's selfishness in his alcoholism and the time that he spends in pubs instead of looking for a solution to his family's financial problems. His depression and strange behaviors make people especially his family resented; however, he never considers others' ideas about himself. He loves alcoholism more than his family in a way that he fights with others to prove his right to drink as much as he desires.

In a word, selfishness is resulted from the aggressiveness of personality. A selfish individual can neurotically be at war with anybody in society and family to obtain his benefits from them. Simon's selfishness in getting a job to afford his life and his family, his unreasonable desire to regain social privileges once he had, and his sense of self-interest push him toward arrogant behaviors. Moreover, Joyce reveals arrogance in Simon to move against others selfishly and become aggressive with them in order to show his prestige in family and society.

## Simon's false sense of power

A false sense of power originates from a radical vanity, which leads individuals to behave irrationally. A false sense of power can be on the base of exaggerated importance of specific skills, possessions, positions, and relationships with powerful people. One way of psychological defense is made by presenting a false sense of power. Another way of a false sense of power can be a denial of powerlessness. According to Horney, it is obvious that aggressive individuals neurotically have a false sense of power. Most of the aggressive neurotics suffer from the lack of power; while they hide their power by exaggerating their powerfulness to others arrogantly.

Obviously, many people go to extremes with their need for power. Several possible reasons for this extremism are: 1) More power can enhance the self-esteem and esteem of others; 2) Those with more power and prestige have a better chance for a brighter future for themselves and their heirs; 3) Power offers better chances for various pleasures; 4) In most societies, the powerful have a greater chance of maintaining and acquiring more power, as the saying goes: “Winner take all and devil take the hindmost.” For many individuals, their possessions and positions of power are important aspects of identity, and the more power they have, the stronger their identity [(15), p. 114].

In *A Portrait of the Artist as a Young Man*, Simon, represented as a strong figure of domination and power, influences the life of his family. His exaggerated need for power moves him against other people. Simon used to be an influential person in the past; however, he is currently in downfall in terms of financial issues and power at home and in society. Simon cannot cope with his reality about his current powerlessness, so he grows to be arrogant, and fights to show his power to others. He tries to affect the people's goals and ideas, especially in the family as “Simon is an anagram for ‘Minos,^1^' it is suggested that in *A Portrait* it is the father who impedes the individual's quest for what he desires, by incarcerating him in a maze of laws” [(5), p. 60]. In his novel, Joyce uncovers Simon's need for power in Simon's wheedling the property owners in order to get rid of paying the rental, in his opinionatedness toward his ideas, and in his desire to control family members.

To begin with, the sense of wheedling emerges as a result of a false sense of power. To wheedle is persuasive or flattering talk with somebody in the hope of getting something in return and having power over them. “Fearless dominance is the part of psychopathy associated with the charming, anxiety-free, and fearless impression that can be used to wheedle a person's way into others' lives and convince them to do things they otherwise wouldn't” (16). Aggressive personalities are skillful at wheedling or obviously supporting others in order to get them to lower their defenses and surrender their loyalty and trust. Aggressive people are also chiefly aware that “people who are to some extent emotionally needy and dependent (and that includes most people who are not character-disordered) want approval, reassurance, and a sense of being valued and needed more than anything” [(17), p. 76]. Appearing to be attentive to these needs can be a wheedler's ticket to incredible power over others. In order to achieve his needs and show how skillful he is, Simon wheedles several people. Simon unfairly wheedles property owners to show his power of convincing others and run away from monthly rentals. At home, these habits get the family from one state of poverty to another, and they have to move houses repeatedly, each time to a less comfortable and less prestigious one: “Goneboro toboro lookboro atboro aboro houseboro; still another removal!” [(8), p. 201]. To add to the financial nightmares of the Dedalus family, Simon has a particular aversion to paying rent for their lodgings. It is the family's custom to stay in one dwelling as long as Simon can wheedle the property owner into letting them stay; then it is off on the increasingly familiar ritual of moving to new quarters. These events follow an almost straight path from good apartments to bad. “Simon Dedalus is never troubled for long by the need to move, for he can always rejoice in his canny ability to find new quarters without paying for the previous ones” [(11), p. 20]. Hence, aggressive people can use any possible way and tool to manipulate others and take the control of their actions and properties. Hence, wheedling is one of the characteristics of aggressive personalities who try to cast their power over others and satisfy their desires. Simon manipulates the property owners in order to solve his rental problems. However, this tactic of Simon is defeated since it is not durable and permanent, and after some time the property owners force them out.

As the second outcome, a false sense of power can cause opinionatedness in personal and social life. Opinionatedness refers to the expression of strong judgments or beliefs about something and showing or having strong and prejudiced opinions. Most aggressive individuals challenge other people in some arrogant ways, even with strong threats. “The aggression may be verbal or physical. Aggressive personalities may scare people and often are identified as too forceful and opinionated” [(18), p. 152]. They behave aggressively in order to show their power and vanity. Opinionated people always verbalize their opinions forcefully without any insight into their influence on others. In *A Portrait of the Artist as a Young Man*, Simon is often in fights with others to force them to accept his ideas, especially on political issues. He tends to turn the table to his side in any debate no matter if he is reasonable or not. He never welcomes any opinions, which are against him. Simon is a person intolerant of any other political and religious opinions. He is an Irish nationalist who always speaks about his political position freely. Moreover, various political arguments make it clear that Simon is a fervent follower of Irish nationalism, particularly of Charles Parnell.^2^ An evident of Simon's opinionatedness happens around Christmas dinner, which is ruined by the serious dispute between Simon and Aunt Dante. Simon leads grace and then a discussion “ensues across the table about the involvement of the Catholic Church in Irish politics centered on the fight for Irish independence, spearheaded by Charles Stuart Parnell and the interjection of the Catholic Church into politics when Parnell's affair with a married woman was discovered” [(19), p. 96]. The quarrel quickly divides the diners. This is both political and gendered. Simon, on one side of the table, leads in voicing his nationalist views; while on the other side, the women, defending the Catholic Church, are positioned in a place of defense. Joyce reflects the power of Simon as he eats hungrily; while Dante and Mary—Simon's wife—lay down their knife and fork unable to eat in the midst of a dispute. Simon's anger on this argument greatly influences Stephen's detached personality who is sitting at the adults' table for the first time at the Christmas dinner. In general, opinionatedness causes a false sense of power, which originates from vanity. Simon struggles to convey his political and religious beliefs aggressively to others. He needs to make sure that everybody follows his ideas obediently. Moreover, Simon as a political person in the past is sensitive to debates about Parnell whose decadence makes Simon jobless and frustrated.

The third outcome of a false sense of power is the desire to dominate other people. When a person or a group has power over others, they are said to have dominance. It is the disposition of a person to assert control in dealing with others. Aggressive people neurotically look for a sense of security by outperforming others, submerging their fears of helplessness and dependence through the constant and unreasonable pursuit of dominance and power. They look for exoneration and refuse self-doubt and criticism. Individuals with a sense of dominance over others enjoy controlling and commanding others in order to practice their power over them. Joyce illustrates Simon as an “angry and embittered man who resents his own family and whose social and financial downfall he blames on others” [(20), p. 50]. Simon shows his dominance by his anger and controlling his family members under his own power. In *A Portrait of the Artist as a Young Man*, Simon's dominance over family members is obvious in his communication with and commanding them. Simon's behavior as a host strengthens his patriarchal position. Asserting the privilege of opening the Charismas dinner, he also assigns seats for the family members. In addition, He commands his son, Stephen, to say the prayer before dinner, besides he “pour[s] sauce freely over Stephen's plate” [(8), p. 26] and seriously lifts the cover of the meal at the time of his own wish. Furthermore, Simon clarifies his power in his interaction with Uncle Charles, which seems more mocking than respectful: “He looked round to where Uncle Charles sat and said: —Now then, there's a bird waiting for you” [(8), p. 25]. The use of “sir” creates a distance between two men, and Simon does know that costly turkey can merely have an impression on the older man. Hence, “while pretending to bond with Uncle Charles through the bantering evocation of feminine “bird,” Simon still maintains the hierarchy between them” [(21), p. 100]. Likewise, Simon confirms his dominance and authority by controlling and reacting to the laughter of Stephen when he is laughing at Simon's joke “—What are you laughing at, you little puppy, you?” [(8), p. 25]. These strange behaviors of Simon make the others detached and ignorant of him in their future interactions and relationships with him. It is understandable from *A Portrait of the Artist as a Young Man* that the false dominance over others is initiated by the need for power and demonstrates an individual's powerlessness. The false impression of dominance eradicates an individual's influence and leads him to aggressiveness. Simon, who gradually loses his power among the family members and even in society, tries to demonstrate his dominance over people by forcing them to behave according to his instruction. He mocks or neglects others simply to prove his sense of higher position in the family and society. The more he mocks, the more others detach from him and leave him aloof.

Thus, aggressive people are anxious about their security and powerlessness in society. They struggle to stay at the top of people's attention by the means of a false sense of power. A hallucinated feeling of power in aggressive people leads them to move against other people and fight for their ambitions mostly irrationally and unfairly. They can prove their power by controlling others through wheedling. Besides, they unveil their power by sticking to their own opinions and forcing others to accept and follow theirs. Finally, aggressive people struggle to be dominant over others' actions and behaviors, which normally results in mocking and belittling others. It is evident that the aggressiveness of Simon helps him find his false sense of power in society and family. Joyce illustrates Simon as an “angry and embittered man who resents his own family and whose social and financial downfall he blames on others” [(20), p. 50]. He uses the power to overcome his insecurity and aloofness in social interactions.

## Simon's search for respect

Sociologically, respect is a positive feeling of esteem for an individual or other entity like a religion, a nation, or also specific actions. Respect is an important component of both personal self-identity and interpersonal relationships. “Feeling respected can almost be considered a basic human right; according regard or perceived worth to someone conveys a sense of respect, and valuing another person's feelings and thoughts is also part of the respect process” [(22), p. 890]. Respect has been studied less than disrespect, with the latter behavior related to stereotyping, prejudice, issues of power and status, injustices, and the like. Disrespecting someone is “serious business and can lead to ruptures in relationships, gang violence, and wars between nations” [(22), p. 891]. While actions that honor something or somebody denote respect, rude behavior is generally considered to suggest a lack of respect. People all look for the esteem of others as a boost to their sense of identity. Neurotic people not only need recognition or basic esteem but also want to be identified as their ideal self, both internally and externally. They need not just for people to tell them they are good but need steady reassurance that they are perfect. Of course, they do not get this, so they are never content with any recognition or respect that they have got. A result of this is that they work hard to obtain respect, which enables them to be successful in some ways, but their extreme desires denote that they will never be satisfied. Simon suffers from a lack of respect. He is not admired by the people in society and the family at his home as much as in the past when his social position and financial situation were both higher than the current fading days. Therefore, the lack of admiration brings about Simon's aggressive personality. He struggles aggressively to force the people in the family and society to respect him. However, his struggle is defeated by others' ignorance and unsatisfactory reactions to his behavior. Joyce divulges Simon's failed attainment of respect from his son, Stephen, and so he is more alienated from his wish to be a gentleman.

The aggressive need for respect is the first reason for a ruined relationship between a father and a son. A lack of respect between a father and his children may lead to a strong sense of detachment and frustration. “Fathers are far more than just ‘second adults' in the home. Involved fathers—especially biological fathers—bring positive benefits to their children that no other person is as likely to bring. They provide protection and economic support and male role models” [(23), p. 151]. Disrespectfulness is one of the improper ways that children may try to react to the aggressiveness of their fathers' behaviors. In *A Portrait of the Artist as a Young Man*, Simon's aggressive personality makes Stephen, alienated from him. However, Simon is eager to get his son's attention and admiration because he is troubled by his sense of insecurity and powerlessness. Simon's aggressiveness makes Stephen openly talk about his father with disrespect and destroy his father's façade. From the beginning of the novel, Stephen's relationship with his father is marked by a certain interpersonal distance. The distance is asserted repeatedly by the look through the glasses, and increases by the time Stephen gets older. “When he is at Clongowes, Stephen pities his father for not being a magistrate like all the other fathers. On the trip to Cork, Stephen already listens to him without sympathy” [(24), p. 29]. Likewise, shortly before Stephen leaves Ireland, he ironically illustrates his father to his friend, Cranly, as “a medical student, an oarsman, a tenor, an amateur actor, a shouting politician, a small landlord, a small investor, a drinker, a good fellow, a storyteller, somebody's secretary, something in a distillery, a tax-gatherer, a bankrupt and at present a praiser of his own past” [(8), p. 274]. Simon's constant aggressiveness and humiliation increase his sense of detachment in Stephen's mind. As a consequence, Simon, who used to be the role model in Stephen's childhood, completely fades out of Stephen's life. It is clear that Simon's weakness in settling up a proper relationship with his son fails. Stephen feels uncomfortable in his father's presence, even embarrassed in the company of others. Besides, the lack of respect in their relationship and Simon's failed fatherhood leave him more arrogant. Hence, it is comprehensible that the aggressiveness and negligence of a father can make children detached. The result of this detachment is the insecurity of the father who will force his children to admire and respect him.

The dishonorableness of a gentleman is the second reason for seeking respect from other people. A gentleman refers to an honorable or courteous man. A true gentleman denotes a man whose behavior includes goodwill and an acute sense of propriety, and whose self-control is equivalent to all emergencies. According to John Wayland, a true gentleman never makes the poor aware of their poverty, “the obscure man of his obscurity, or any man of his inferiority or deformity; who is himself humbled if necessity compels him to humble another; who does not flatter wealth, cringe before power, or boast of his own possessions or achievements” [qtd. in Glass (25), p. 201]. In *A Portrait of the Artist as a Young Man*, Simon struggles to be and behave like a gentleman in order to get respect and admiration from the people around him. However, his arrogant behaviors and overstated vanity make him away from the attributes of a gentleman. Similarly, aggression removes the air of a gentleman in social interactions and personal life. Simon considers himself a gentleman. A gentleman, following Richard Allestree's definition, “possesses wealth, authority, education, reputation and time, which gives him the opportunity to dedicate his life to leisure activities without the need to work himself” [(26), p. 104]. Simon's time of prosperity was before 1860 and, therefore, we currently find Simon as a frustrated person who is often living in nostalgia for old times. “Simon Dedalus' characterization concentrates on his outward appearance and his representation in public. We learn that he has a beautiful singing voice, is sociable, and takes care of his outward appearance” [(24), p. 17]. Thereby he comes across as a hollow effigy, a mere echo of his former importance. On the trip to Cork, his hollowness is intensified by the absence of any of his fellow students who are all dead then. “Simon Dedalus feverishly tries to evoke his past by presenting “evidence” of his youth. He fails in his attempt to bring his own youth to life in order to impress his son. Moreover, it has the opposite effect and adds to his financial decline” [(24), p. 42].

Similarly, the lack of a sense of an honorable gentleman also appears in the family. At home, in a scene, Simon asks, “Is your lazy bitch of a brother gone out yet?” [(8), p. 198] His rude words demonstrate that he is anything but gentlemanly. Generally, the lack of virtues of a gentleman clarifies is shown in his arrogance and rudeness. Simon used to be a true gentleman in the past when his financial and social status was highly admirable. However, he has lost his traits of a gentleman along with his properties and fame. Simon is unable to cope with his current situation, which leads him to aggression and struggle to force people to admire him both in society and at home. As a result, Simon moves against people seriously in order to reclaim his respect, and overcome his sense of insecurity.

At last, a strong sense of antifemininity can seriously damage the respectful relationship between a man and woman. Antifemininity implies the opposition or discrimination against women or feminine characteristics and behaviors. “Antifemininity encourages men to see themselves as having nothing in common with women. It also encourages men to view feminine traits and females as less valuable than masculine traits and males” [(27), p. 53]. Therefore, Antifemininity results in disrespect for women and inhibits the formation of men's relationships with women as well as equal partner types of romantic relationships. Moreover, numerous culturally defined feminine skills and traits have significant implications for quality of life; however, most men avoid taking on these traits and learning these skills since they are afraid of being considered unmasculine. In order to be respected, men need to get the attention of women. Disrespecting any gender can result in detachment and aloofness. Simon needs others' admiration to get rid of his insecurity and anxieties. However, he has a disrespectful and ignoring viewpoint toward females. To overcome the hostility of femininity, Simon becomes aggressive in behavior. Joyce uncovers the feeling of antifemininity in Simon from the beginning of the novel. When having a Christmas dinner with his family and friends, Simon forgets to offer Dante sauce for her meal, “a negligence which is criticized by his wife” [(8), p. 26]. Her effort to correct Simon's mistake is, however, blocked by Dante with a brief “No thanks” [(8), p. 26]. In contrast, he is very careful with his friend Casey's dinner. Moreover, he is having his dinner without showing concern for the females around the table. Simon's formal time to spend communicating with females is when he is heavily drunk at a bar chatting with barmaids unconsciously. The distorted sense of femininity and vanity over them makes his wife and female acquaintances detached from him. Generally, the lack of admiration in an individual's personality and behavior toward a specific gender can render the aggressiveness of that person. Simon as the head of the family has improper behavior with females in the family. On the one hand, Simon does not control his aggressiveness; on the other hand, he needs the people's respect in order to keep his face. Therefore, failing to gain the respect of the females, Simon arrogantly experiences coldness and frustration due to his false vanity and overstated self-esteem against femininity. Failure in obtaining other people's respect results in inappropriate behaviors with Simon's family members, ruin his wishes, and cause negative reactions to the opposite gender.

## Conclusion

In conclusion, having or revealing an exaggerated sense of one's own importance or abilities cause arrogance and vanity and bring about neurotic personality. As said, the need for power, exploitation, Prestige, admiration, and achievement shape neurotic personality. In James Joyce's *A Portrait of the Artist as a Young Man*, Simon is in need of admiration; while he has a wrong struggle to attain it. He behaves with his son aggressively, which ends in his son's detachment from him. Moreover, he ruins the ideals he had created for his self in the past. Lastly, he has indecent behaviors with females at home and in society. All these offensive conducts make him more insecure and leave him more aggressive toward everybody. This aggressive personality makes him neurotically move against people in society and family.

## Data availability statement

The original contributions presented in the study are included in the article/supplementary material, further inquiries can be directed to the corresponding author.

## Author contributions

All authors listed have made a substantial, direct, and intellectual contribution to the work and approved it for publication.

## Footnotes

^1^Minos, in Greek mythology, is the first King of Crete, son of Europa and Zeus. Every nine years, Minos makes King Aegeus choose seven young girls and seven young boys to be conveyed to Daedalus's creation, the labyrinth, to be eaten by the Minotaur. Minos, after his death, becomes a judge of the dead in the underworld.

^2^Charles Stewart Parnell (1846–1891) is an Irish Nationalist, a member of the British Parliament (1875–91), and the leader of the struggle for Irish Home Rule in the late 19th century. He makes Home Rule for Ireland a major factor in Irish nationalism and British politics.
